# Supplementary material for: A Thermosensitive, Chitosan-Based Hydrogel as Delivery System for Antibacterial Liposomes to Surgical Site Infections
Source: Pharmaceutics. 2022 Dec 18;14(12):2841. doi: 10.3390/pharmaceutics14122841 (PMC9784289; doi:10.3390/pharmaceutics14122841)
Supplement: Supplementary file 1 [file pharmaceutics-14-02841-s001.zip › pharmaceutics-2082153-supplementary.pdf]

Supplementary file

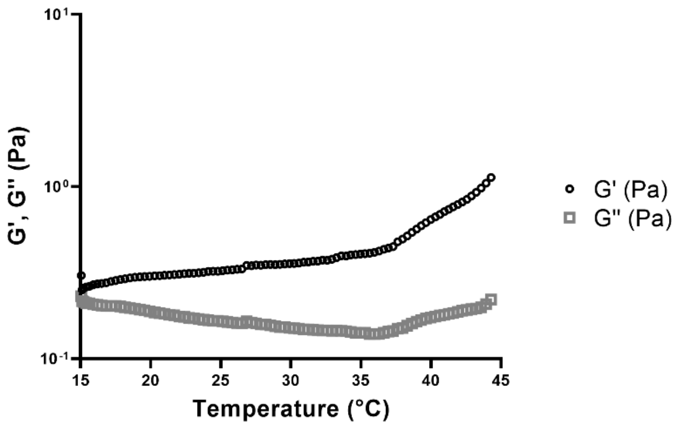

Supplementary Figure S1. Representative rheological curves of temperature-dependent sol-gel transition of autoclaved chitosan with sterile filtered β-glycerophosphate (CS-βGP; molar ratio 1:4.88). Measurement of the storage modulus (G') and loss modulus (G'') during a temperature range.
